# Shared genetic factors and causal association between chronic hepatitis C infection and diffuse large B cell lymphoma

**DOI:** 10.1186/s13027-024-00577-4

**Published:** 2024-04-23

**Authors:** Leihua Fu, Jieni Yu, Zhe Chen, Feidan Gao, Zhijian Zhang, Jiaping Fu, Weiying Feng, Pan Hong, Jing Jin

**Affiliations:** Department of Hematology, Shaoxing People’s Hospital, 312000 Shaoxing City, Zhejiang Province China

**Keywords:** Chronic Hepatitis C virus, Diffuse large B cell lymphoma, Mendelian randomization, Colocalization analysis

## Abstract

**Background:**

Epidemiological research and systematic meta-analyses indicate a higher risk of B-cell lymphomas in patients with chronic hepatitis C virus (HCV) compared to non-infected individuals. However, the genetic links between HCV and these lymphomas remain under-researched.

**Methods:**

Mendelian randomization analysis was employed to explore the association between chronic hepatitis C (CHC) and B-cell lymphomas as well as chronic lymphocytic leukemia (CLL). Approximate Bayes Factor (ABF) localization analysis was conducted to find shared genetic variants that might connect CHC with B-cell lymphomas and chronic lymphocytic leukemia (CLL). Furthermore, The Variant Effect Predictor (VEP) was utilized to annotate the functional effects of the identified genetic variants.

**Results:**

Mendelian randomization revealed a significant association between CHC and increased diffuse large B cell lymphoma (DLBCL) risk (OR: 1.34; 95% CI: 1.01–1.78; *P* = 0.0397). Subsequent colocalization analysis pinpointed two noteworthy variants, rs17208853 (chr6:32408583) and rs482759 (chr6:32227240) between these two traits. The annotation of these variants through the VEP revealed their respective associations with the butyrophilin-like protein 2 (BTNL2) and notch receptor 4 (NOTCH4) genes, along with the long non-coding RNA (lncRNA) TSBP1-AS1.

**Conclusion:**

This research provides a refined genetic understanding of the CHC-DLBCL connection, opening avenues for targeted therapeutic research and intervention.

**Supplementary Information:**

The online version contains supplementary material available at 10.1186/s13027-024-00577-4.

## Introduction

About 16% of human cancers are linked to infectious agents, predominantly due to viral and bacterial infections [1]. Chronic hepatitis C (CHC) virus infection, primarily affecting the liver, also poses systemic risks, significantly increasing the likelihood of developing B-cell lymphomas [2]. Extensive research, including comprehensive studies and meta-analyses, has underscored an elevated risk of B-cell non-hodgkin’s lymphoma in patients with CHC virus compared to those without the infection [3, 4].

Pathophysiological processes from hepatitis C virus (HCV) infection to overt lymphoma involve mechanisms like sustained antigenic stimulation leading to monoclonal expansion and direct viral roles in cellular transformation, especially in large B-cell lymphoma [5, 6]. In vitro studies indicate that the CD81 receptor, crucial for HCV entry, plays a role in B cell infection [7]. HCV replication in B cells might initiate oncogenic events through intracellular viral proteins [8]. This process is linked to oxidative stress caused by viral proteins core and NS3, potentially leading to DNA mutations and repair abnormalities, resulting in cell transformation [9]. Additionally, genomic mutations are also implicated in this process, supported by identified mutations in Neurogenic Locus Notch Homolog Protein (NOTCH) 2, NOTCH1, and Phosphatase and Tensin Homolog (PTEN) in HCV-positive diffuse large B cell lymphoma (DLBCL) patients [10]. However, the molecular pathology linking HCV infection with lymphoma remains elusive. Advanced studies are essential for a comprehensive understanding of the genetic interplay in HCV-associated B-cell lymphoma [3].

Our research utilizes Genome-Wide Association Studies (GWAS) data for an advanced Mendelian randomization (MR) analysis [11–14], a robust method that identifies causal relationships between risk factors and health outcomes while effectively minimizing confounding influences, to establish a causal link between CHC virus infection and B-cell lymphomas as well as chronic lymphocytic leukemia (CLL). We further employ colocalization analysis [15], which plays a pivotal role in uncovering shared molecular mechanisms underlying various diseases and their associated intermediate phenotypes, to discern the causal variants that underlie this association, potentially elucidating the genetic interplay between these conditions. This approach is expected to yield advanced insights into the genetic underpinnings of the relationship between CHC virus infection and B-cell lymphoproliferative disorders.

## Materials and methods

### Study design

**Fig. 1:**
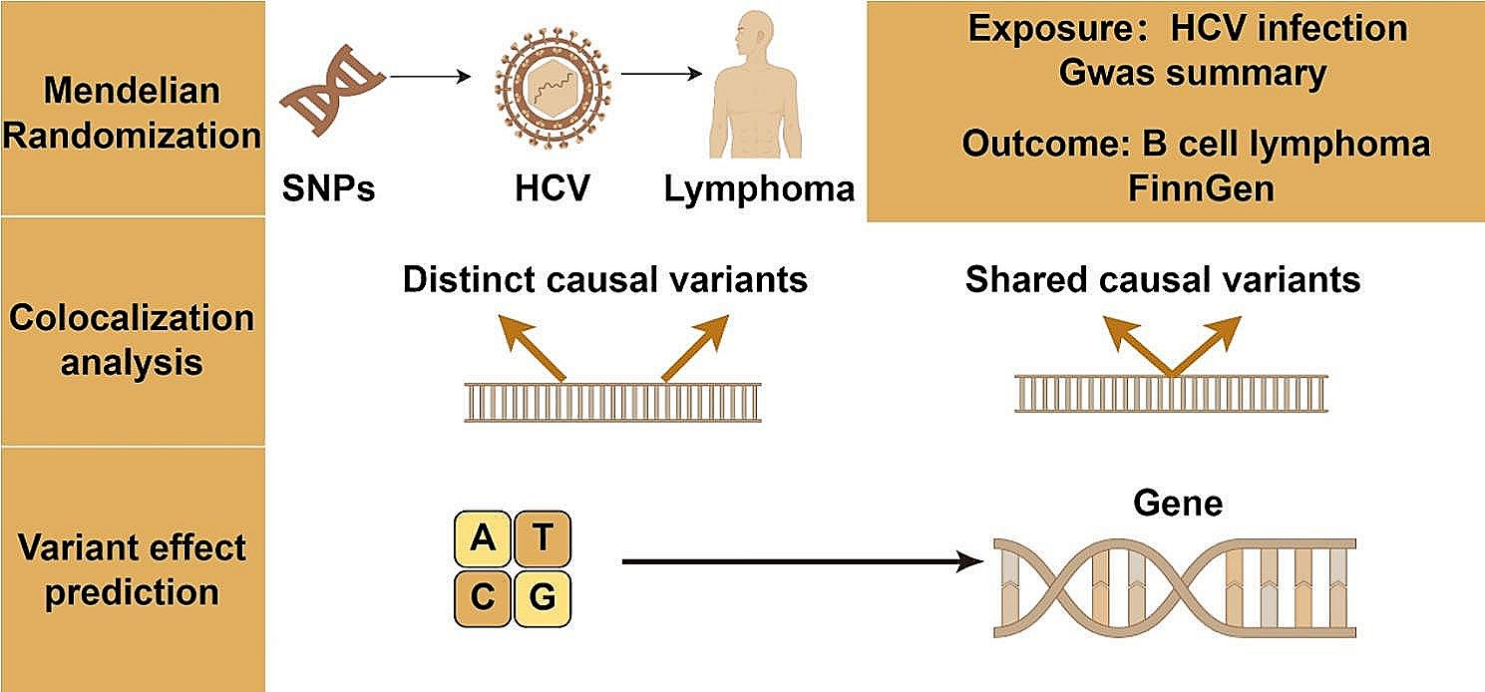
A detailed flowchart showing the steps of the study

Figure 1 delineates the comprehensive study design. In the initial phase, the study leverages a MR framework, utilizing genetically derived instrumental variables (IVs) to robustly infer the causal nexus between CHC and B-cell lymphomas, inclusive of CLL. Subsequently, in the event of establishing a causal linkage between CHC and other traits, colocalization analysis is employed to discern shared causal genetic variants underpinning CHC and these traits. The final stage involves the application of advanced gene annotation methodologies to prognosticate the consequent effects on genomic and proteomic functionalities.

### Data sources

We obtained CHC-related data for the European population (*N* = 352,013) from the IEU Open GWAS project and selected SNPs associated with CHC as IVs from the GWAS summary database (https://gwas.mrcieu.ac.uk/). From the Finnish database (https://www.finngen.fi/en), we downloaded various types of B-cell lymphoma outcome data, including DLBCL (*N* = 315,243), marginal zone lymphoma (MZL) (*N* = 314,395), mantle cell lymphoma (MCL) (*N* = 314,403), Waldenström’s macroglobulinemia (WM) (*N* = 314,281), and CLL(*N* = 314,857) as well. Detailed data information is provided in Supplementary Data (Tab. S1).

### Selection of instrumental variables

In our approach, we set a genome-wide significance threshold at *P* < 5 × 10^− 8^ for identifying SNPs strongly correlated with CHC. To select independent SNPs and reduce the influence of linkage disequilibrium (LD), we applied a threshold of 0.01 for the LD parameter (r^2^) and a genetic distance criterion of 1000 kb (r^2^ < 0.01 and clump distance = 1000 kb), based on the European 1000 Genomes Project reference panel. The association strength between the instrumental variables and exposure factors was evaluated using the *F* statistic. To avoid bias from weak instrumental variables, our criteria included only SNPs with an *F* statistic greater than 10 [16, 17]. Detailed data information is provided in Supplementary Data (Tab. S2).

### Mendelian randomization

In our study, we conducted a two-sample MR analysis to explore the association between CHC and B-cell lymphomas, as well as CLL, utilizing the “TwoSampleMR” package (Version 0.5.8) in R software. The MR analysis incorporated several methods, notably the inverse variance-weighted model (IVW), MR-Egger, and the weighted median approach [18–21]. IVW was the primary analytical method, with MR-Egger and the weighted median serving as complementary techniques to enhance the reliability and robustness of our findings [21].

To validate our findings on the causal relationship between CHC and B-cell lymphomas, including CLL, we executed a series of sensitivity analyses. We assessed heterogeneity using Cochran’s Q statistic in both the IVW and MR-Egger methods [22], considering a p-value over 0.05 as indicative of non-significant heterogeneity [20]. Additionally, to test for horizontal pleiotropy, we employed the Egger intercept and MR-PRESSO method [20], with a p-value above 0.05 suggesting no evidence of horizontal pleiotropy.

### Colocalization analysis

We conducted an approximate Bayes factor (ABF) localization analysis to investigate potential shared genetic causal variants in gene regions that may link CHC, B-cell lymphomas, and CLL, utilizing the “COLOC” package (Version 5.2.3) in R software.

ABF localization analysis have five hypotheses [15]:

#### Hypothesis 0 (H0):

Neither Trait 1 nor Trait 2 is associated with the genetic variant.

#### Hypothesis 1 (H1):

Only Trait 1 is associated with the genetic variant.

#### Hypothesis 2 (H2):

Only Trait 2 is associated with the genetic variant.

#### Hypothesis 3 (H3):

Both traits are associated with the genetic variant, but each association is independent of the other.

#### Hypothesis 4 (H4):

Both traits are associated with the genetic variant due to a shared causal mechanism.

To perform this colocalization analysis, we created windows of ± 20 kb around the SNPs used for CHC trait IVs. We then executed ABF analysis using standard settings to calculate the posterior probability of the CHC, B-cell lymphomas and CLL having a shared causal signal (under Hypothesis 4: both traits are influenced by a common causal variant in the gene region). By standard practice, a posterior probability for H4 (PP.H4) > 85% was regarded as indicative of colocalization [15]. This suggests the presence of a causal genetic variant linking CHC, B cell lymphoma and CLL, possibly pointing to a shared genetic basis in their association.

### Variant effect prediction

Utilizing the Variant Effect Predictor (VEP) tool (https://asia.ensembl.org/Tools/VEP) for functional annotation of causal variants identified through colocalization analysis between CHC, B-cell lymphomas and CLL [23].

## Results

### Causal effect on B cell lymphomas and CLL

Upon screening, five independent SNPs linked to CHC were identified, with *F*-statistics ranging from 33.61 to 92.94. This range suggests a reduced probability of bias due to weak instrumental variables.

**Fig. 2:**
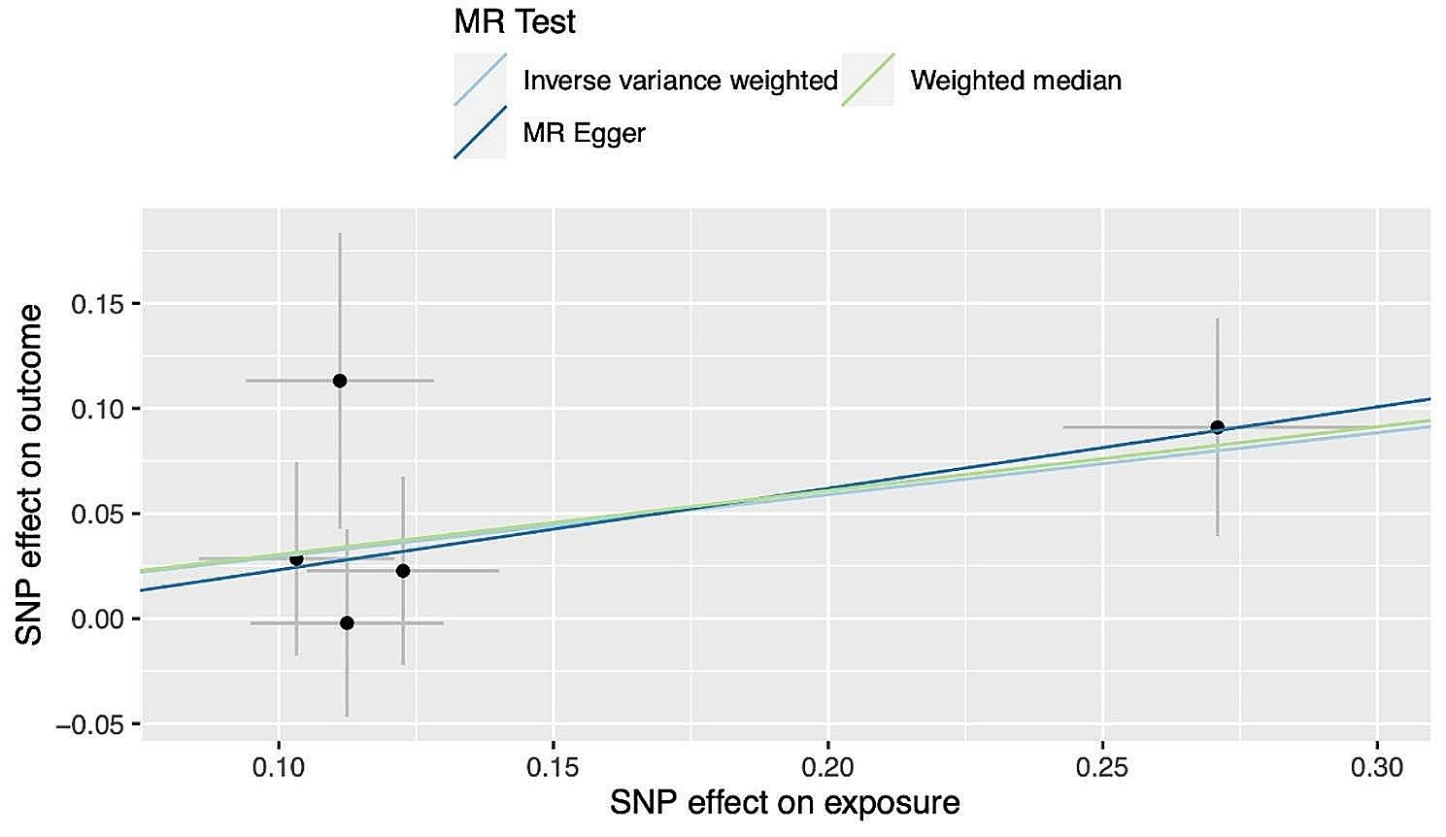
Scatter plots for causal association between CHC and DLBCL

Subsequently, a Two Sample MR analysis was conducted to assess the causal relationship between CHC and various B cell lymphoma subtypes, including DLBCL, MCL, MZL, WM, as well as CLL. The primary outcome, derived via IVW method, indicated a causal association between CHC infection and DLBCL (OR: 1.34; 95% CI: 1.01–1.78; *P* = 0.0397) (Fig. 2). However, our analysis did not reveal similar causal associations for CHC with other subtypes of B cell lymphoma and CLL.

**Fig. 3:**
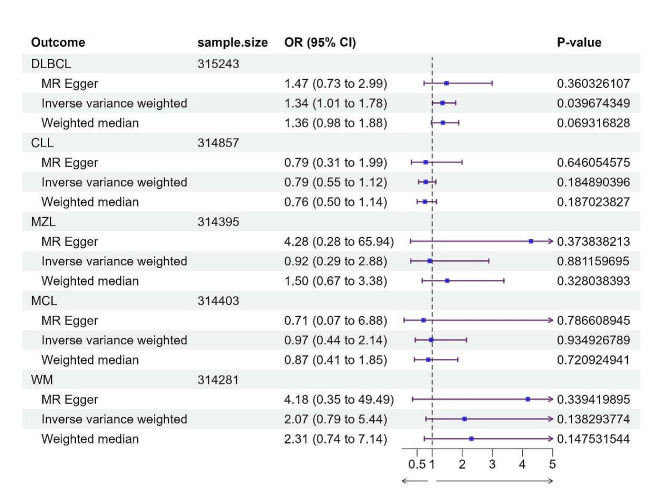
The forest plot, utilizing IVW, MR-Egger, and weighted median methods, demonstrated causal associations between CHC and Bcell lymphomas, including CLL. A significant causal relationship was found between CHC infection and DLBCL (OR: 1.34; 95% CI: 1.01–1.78; *P* = 0.0397)

Comprehensive sensitivity analyses, including heterogeneity assessment using Cochran’s Q test, revealed significant heterogeneity from CHC to MZL (Q-value = 0.0124, I2 = 68.72%). Consequently, a random effects IVW model was employed to estimate the MR effect size for this relationship. In contrast, analyses of other lymphoma subtypes did not exhibit such heterogeneity. Further, horizontal pleiotropy was evaluated using MR-Egger regression, and supplementary MR-PRESSO testing did not reveal any data outliers (Fig. 3, Supplementary Data Tab. S3).

### Colocalization analyses of CHC with DLBCL

**Fig. 4:**
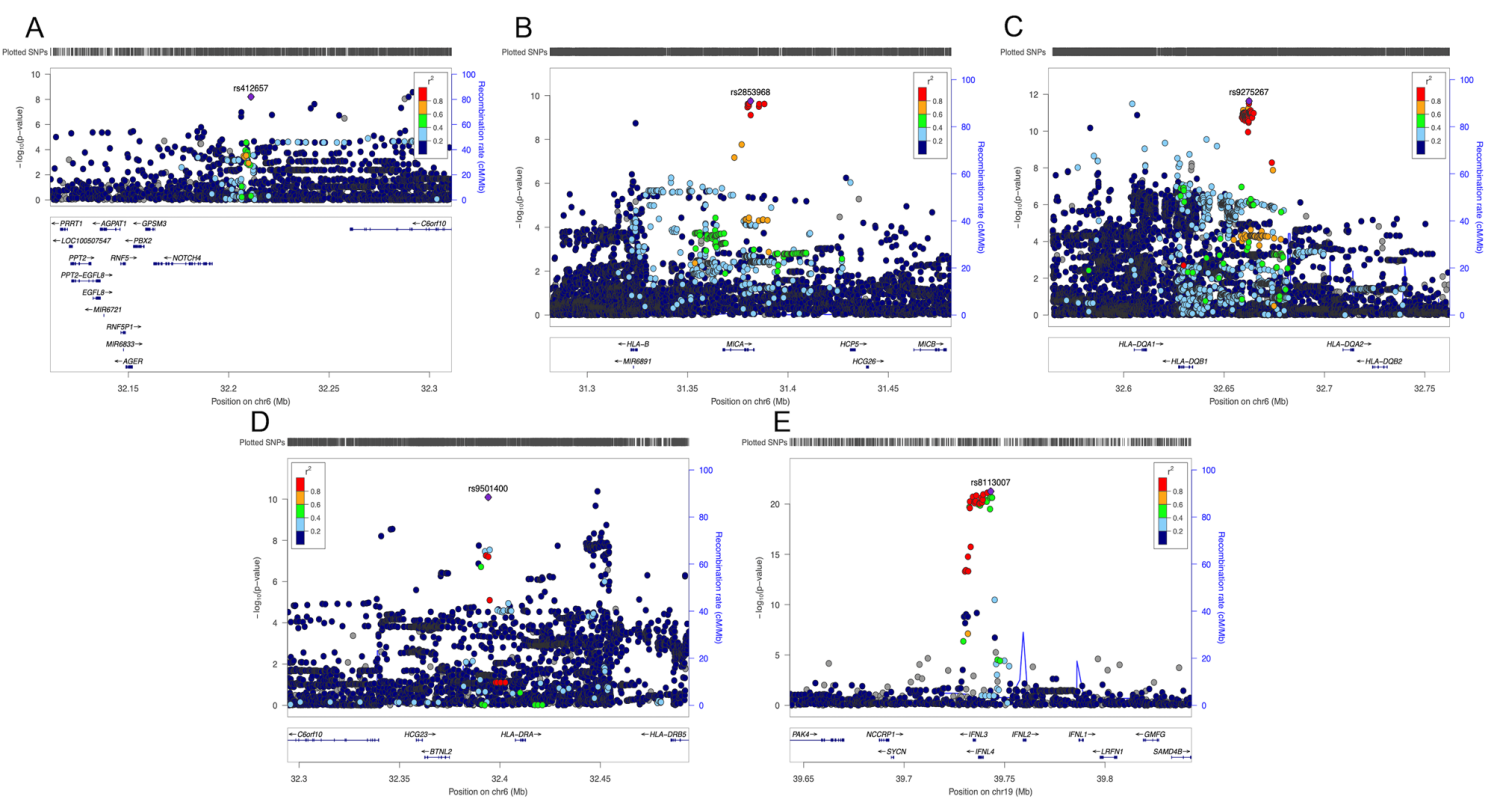
The LocusZoom plot in the study displays the SNPs used for analyzing the CHC trait, featuring five key variants located on chromosomes 6 and 19. The plot displays -log10 *P*-value on the y-axis and physical position on the x-axis. Points identify individual variants whose colour indicates their LD r2 with key variants. A: rs412657(chr6: 32211085), B: rs2853968(chr6:31381351), C: rs9275267 (chr6:32662425). D: rs9501400(chr6:32394184). E: rs8113007(chr19: 39743103)

**Fig. 5:**
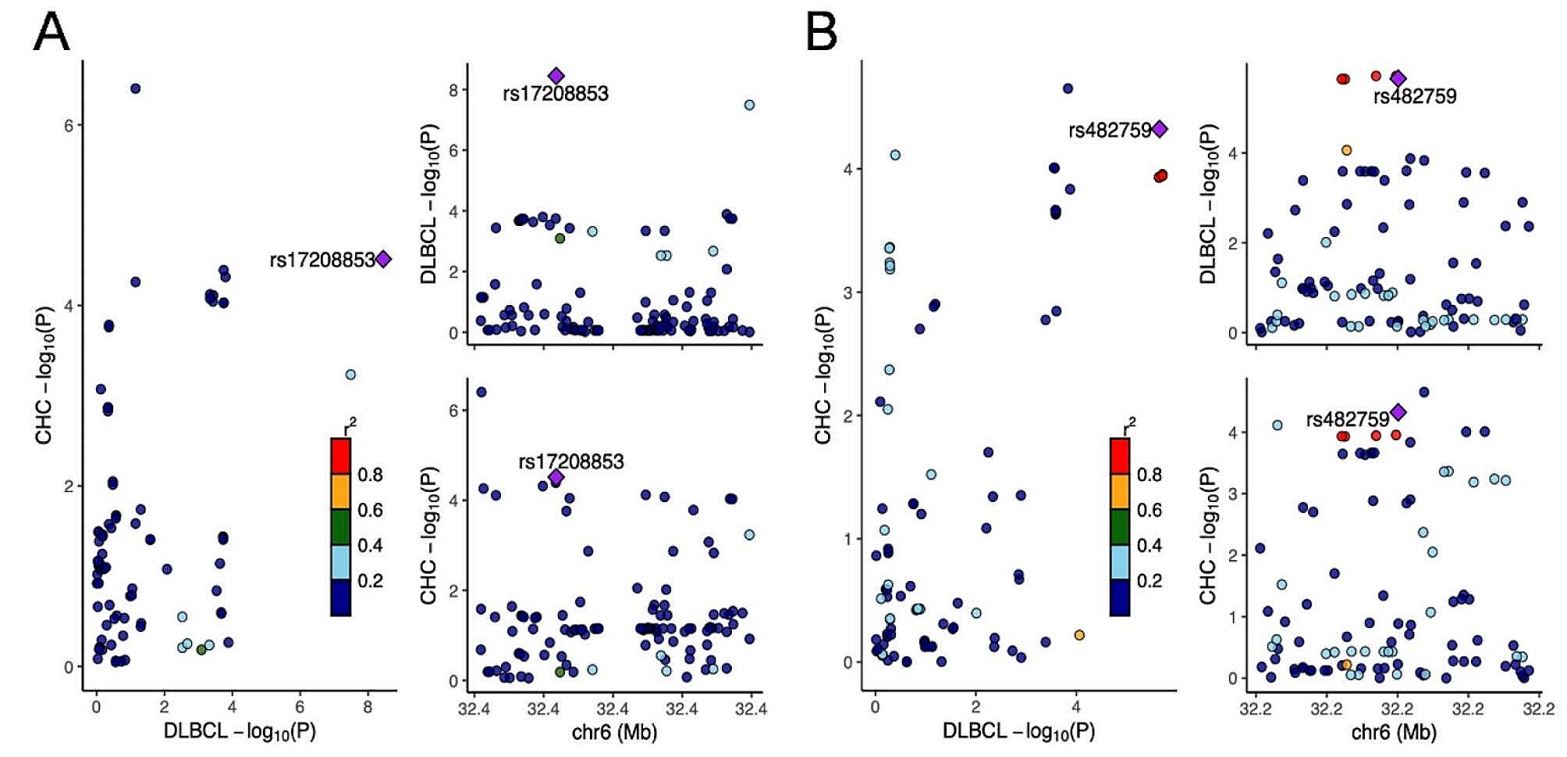
Colocalization analysis results for the association between CHC and DLBCL. A: A shared causal variant within the ± 20 kb gene region of rs9501400, which leads to the causal variant rs17208853. B: A shared causal variant within the ± 20 kb gene region of rs412657, which leads to the causal variant rs482759

To further investigate the potential causal variant linking CHC and DLBCL, a colocalization analysis was conducted in gene regions for these traits (Fig. 4). The analysis indicated a high posterior probability (94.00% PP.H4) of a shared causal variant within the ± 20 kb gene region of rs9501400 (chr6: 32394184), which leads to the causal variant rs17208853. Additionally, a similar high posterior probability of 93.40% PP.H4 was ascertained for the sharing of a causal variant within the gene region (± 20 kb) of rs412657 (chr6: 32211085), culminating in the causal variant rs482759. And notably, In the chr6:32642425–32682425 region, our analysis revealed that CHC and DLBCL share independent variants with a high posterior probability of 99.90% PP.H3, indicating distinct genetic influences in this specific genomic area (Fig. 5; Table 1).

**Table 1:** The result from colocalization analysis of chronic hepatitis C infection with DLBCL

SNPs	CHR	Position	Region	PP.H3	PP.H4	Best causal	SNP.PP.H4
rs2853968	6	31381351	Chr6:31361351–31401351	0.707%	2.200%	NA	NA
rs9275267	6	32662425	Chr6:32642425–32682425	99.900%	0.021%	NA	NA
rs9501400	6	32394184	Chr6:32374184–32414184	4.570%	94.000%	rs17208853	0.9918
rs412657	6	32211085	Chr6:32191085–32231085	1.280%	93.400%	rs482759	0.3506
rs8113007	19	39743103	Chr19:39723103–39763103	0.000%	0.001%	NA	NA

### Variant effect prediction

Utilizing the Variant effect prediction (VEP) online tool, we executed a rigorous annotation of SNPs rs482759 and rs17208853. The results designate rs482759 as an upstream regulatory variant for NOTCH4, involved in protein coding modulation and intronic sequence maintenance. Simultaneously, rs17208853, mapped upstream of butyrophilin-like protein 2(BTNL2) gene, emerges as a multifunctional regulatory agent, influencing both protein-coding and non-coding RNA transcripts, including those marked for nonsense-mediated decay. This highlights its intricate regulatory capacity in BTNL2 gene expression. Additionally, The SNP rs17208853, located downstream of the TSBP1 and BTNL2 antisense RNA 1(TSBP1-AS1), is classified as a modifier and may impact the expression and regulatory mechanisms of this lncRNA (Supplementary Data Tab. S4).

## Discussion

In our own study, we employed MR to investigate the association between CHC and B-cell lymphoma subtypes, along with CLL. Our findings indicated a statistically significant elevation in the risk of DLBCL associated with CHC infection (OR: 1.34; 95% CI: 1.01–1.78; *P* = 0.0397). This evidence corroborates the causal inference drawn from prior observational correlations [24–27]. Additionally, previous systematic meta-analysis found that, besides DLBCL, CHC is also associated with other types of B-cell lymphoma such as MZL, lymphoplasmacytic lymphoma (LPL), and follicular lymphoma (FL) [25, 28–30]. However, our study did not establish a causal relationship between CHC and these B-cell lymphoma subtypes as well as CLL. Several factors could account for this observed discrepancy. Firstly, MR utilizes genetic variants as IVs to investigate the causal relationship between exposure and outcome, with a critical assumption being the exclusion of confounders [11, 12]. For instance, during the selection of the exposure-related IVs, factors such as chronic inflammatory stimuli are intentionally excluded as confounders, given that chronic inflammation is a recognized pathogenic mechanism for lymphoma [31]. In other words, a known contributing factor to lymphoma pathogenesis is deliberately eliminated. Secondly, the pathogenesis of CHC-related lymphoma involves a complex cascade of molecular and cellular events. The development of HCV-related lymphoma involves ongoing stimulation of lymphocytes by viral antigens, leading to cell proliferation; oncogenic effects from HCV replication in B cells, mediated by viral proteins; and lasting B-cell damage from transient viral presence, a phenomenon known as the “hit and run” theory [32]. Nevertheless, these critical pathogenic factors are not assessed in MR studies. In a systematic meta-analysis [33, 34], however, the calculated incidence rate of lymphoma accounts for the possibility of all occurrences of the disease. The exclusion or infeasibility of evaluating these factors may result in MR being less capable to provide as comprehensive and conclusive a result as a systematic meta-analysis might, potentially contributing to the discrepancy between their findings. The last potential reason for inconsistencies might be inadequate statistical power in MR studies, influenced by the prevalence of genetic variants used, their effect size on the risk factor, and the study sample size [12, 14]. These factors may lead to discrepancies between the conclusions of epidemiological studies and those derived from MR, yet the two sets of findings are not necessarily contradictory.

Recent advancements have decrypted various pathogenic pathways linking CHC to non-Hodgkin lymphoma, emphasizing the significant role of genetic determinants in HCV-associated lymphoproliferative disorders [9, 10, 35, 36]. In a comprehensive French study with 87 HCV-associated lymphoma patients, the TNF alpha induced protein 3 (TNFAIP3)/A20 gene’s rs2230926G allele was found more frequently in patients exhibiting rheumatoid factor (RF) activity (20%). This correlation suggests that even minimal A20 dysfunction, potentially resulting in heightened NF-kB activity, might be enough to trigger the lymphomatous transformation in autoimmune B cells, particularly under conditions of chronic RF + B cell stimulation [37]. In our study, we utilized colocalization analysis [15], which plays a pivotal role in uncovering shared molecular mechanisms underlying various diseases and their associated intermediate phenotypes, we pinpointed two causal variants - rs17208853 and rs482759 - that mediate the correlation between CHC infection and DLBCL, implying a potential shared genetic underpinning for these two traits.

Further VEP analysis suggested that rs17208853 and rs482759 are regulatory variants affecting BTNL2 and NOTCH4genes, with rs17208853 also influencing the lncRNA profile of TSBP1-AS1. The BTNL2 gene functions predominantly as an immune regulator, with high expression in lymphoid tissues and structural homology to B7 co-stimulatory molecules [38, 39]. It is implicated in dampening T-cell proliferation and cytokine production, thereby modulating T-cell-mediated responses, especially in the gastrointestinal tract [40]. Research by Waller RG et al. recognizes BTNL2 among genes that elevate the risk of multiple myeloma, indicating potential genetic overlap with other lymphoid malignancies [41]. Additionally, Vijai J et al. identify loci near BTNL2 linked to MZL, emphasizing its role in lymphoma pathogenesis [42]. Although no direct genetic link between CHC and MZL was found in our study, it is important to note that MZL carries a risk of transformation into DLBCL [43]. The NOTCH pathway is recurrently mutated in DLBCL associated with hepatitis C virus infection [10]. NOTCH2, NOTCH1 and PTEN mutations have been identified in respectively, 20%, 4% and 2% of HCV-positive DLBCL patients [10]. NOTCH4, a key component of the NOTCH pathway, has been shown to have significant associations with both benign and malignant lymphoproliferative diseases related to HCV [44, 45]. Our colocalization analysis has identified rs482759 as a regulatory variant located upstream of the NOTCH4 gene. This variant is involved in the encoding of NOTCH4 proteins and may play a role in the pathogenesis of DLBCL related to CHC. The TSBP1-AS1 gene, spanning 139 kb and characterized as a lncRNA, is highly expressed in immune system cells [46]. It is notable for its partial overlap with the protein-coding genes TSBP1 (also known as C6orf10) and BTNL2, which are transcribed from the opposite strand [47, 48], and have pleiotropic effects on autoimmune disorders [49, 50]. The precise function of TSBP1-AS1 in lymphomas remains to be elucidated, necessitating further focused research to unravel its potential roles in lymphomagenesis.

Our research constitutes a pioneering application of MR to investigate the risk association between CHC and B-cell proliferative diseases, establishing CHC as a risk factor for DLBCL and identifying two causal genetic variants. This contributes significantly to understanding the genetic interplay between these pathologies. However, our results also have some limitations. Firstly, the study’s concentration on a European population limits the extension of our findings to other ethnic groups. Additionally, despite the identification of a colocalized signal, the complex underlying genetic mechanisms still require more comprehensive exploration. Furthermore, our analysis, while comprehensive for various B-cell lymphoma subtypes, faced limitations due to data availability, which restricted the inclusion and examination of certain lymphomas, such as FL.

## Conclusion

In summary, our study utilizes Mendelian randomization to explore the genetic relationship between CHC and B-cell lymphoproliferative disorders, particularly focusing on DLBCL. We successfully identified CHC as a contributing risk factor for DLBCL and uncovers specific genetic variants that provide a causal link. These findings offer significant insights into the genetic interplay between these conditions. Further clinical and experimental studies are required to validate the relationship between CHC and B-cell lymphoproliferative disorders.

### Electronic supplementary material

Below is the link to the electronic supplementary material.

Supplementary Material 1

Supplementary Material 2

Supplementary Material 3

Supplementary Material 4

## Data Availability

Only publicly available data were used in this study, and data sources and handling of these data are described in the Methods and Supplementary Data 1–4. Chronic hepatitis C infection data could be obtained from GWAS summary database (https://gwas.mrcieu.ac.uk/). B cell lymphomas and chronic lymphocytic leukemia could be obtained from Finnish database (https://www.finngen.fi/en). Further information is available from the corresponding author upon request.

## Abbreviations

CHC: Chronic hepatitis C
HCV: Hepatitis C virus
NOTCH: Neurogenic Locus Notch Homolog Protein
PTEN: Phosphatase and Tensin Homolog
DLBCL: Diffuse large B cell lymphoma
GWAS: Genome-Wide Association Studies
MR: Mendelian randomization
CLL: Chronic lymphocytic leukemia
IVs: Instrumental variables
MZL: Marginal zone lymphoma
MCL: Mantle cell lymphoma
WM: Waldenström's macroglobulinemia
LD: Linkage disequilibrium
IVW: Inverse variance-weighted model
ABF: Approximate Bayes factor
VEP: Variant Effect Predictor
BTNL2: Butyrophilin-like protein 2
TSBP1-AS1: TSBP1 and BTNL2 antisense RNA 1
LPL: Lymphoplasmacytic lymphoma
FL: Follicular lymphoma
TNFAIP3: TNF alpha induced protein 3
RF: Rheumatoid factor
